# Musashi1 regulates breast tumor cell proliferation and is a prognostic indicator of poor survival

**DOI:** 10.1186/1476-4598-9-221

**Published:** 2010-08-21

**Authors:** Xiao-Yang Wang, Luiz OF Penalva, Hongyan Yuan, R Ilona Linnoila, Jiachun Lu, Hideyuki Okano, Robert I Glazer

**Affiliations:** 1 Department of Oncology, Georgetown University, and Lombardi Comprehensive Cancer Center, Washington, DC 20007, USA; 2 Cell and Cancer Biology Branch, Center for Cancer Research, National Cancer Institute, National Institutes of Health, Bethesda, MD 20892, USA; 3 Department of Cell and Structural Biology, University of Texas Health Science Center at San Antonio, TX, USA; 4 Department of Epidemiology, State Key Lab of Respiratory Disease, Guangzhou Medical College, Guangzhou, Guangdong, China; 5 Department of Physiology, Keio University School of Medicine, Tokyo, Japan

## Abstract

**Background:**

Musashi1 (Msi1) is a conserved RNA-binding protein that regulates the Notch and Wnt pathways, and serves as a stem cell marker in the breast and other tissues. It is unknown how Msi1 relates to other breast cancer markers, whether it denotes tumor initiating cells (TICs), and how it affects gene expression and tumor cell survival in breast cancer cells.

**Results:**

Msi1 expression was analyzed in 20 breast cancer cell lines and in 140 primary breast tumors by western blotting and immunohistochemistry, respectively. Lentivirus RNA interference was used to reduce Msi1 expression in breast cancer cell lines MCF-7 and T47D grown as spheroid cultures and to assess stem cell gene expression and the growth of these cell lines as xenografts. In normal human breast tissue, Msi1 was expressed in 10.6% of myoepithelum and 1.2% of ductal epithelium in the terminal ductal lobular unit (TDLU), whereas, less than 0.05% of ductal epithelium and myoepithelium in large ducts outside the TDLU expressed Msi1. Msi1 was expressed in 55% of the breast cancer cell lines and correlated with ErbB2 expression in 50% of the cell lines. Msi1 was expressed in 68% of primary tumors and in 100% of lymph node metastases, and correlated with 5 year survival. Msi1 was enriched in CD133^+ ^MCF-7 and T47D cells and in spheroid cultures of these cells, and Msi1 'knockdown' (KD) with a lentivirus-expressed shRNA decreased the number and size of spheroid colonies. Msi1 KD reduced Notch1, c-Myc, ErbB2 and pERK1/2 expression, and increased p21^CIP1 ^expression, which is consistent with known Msi1 target mRNAs. Msi1 KD also reduced the expression of the somatic and embryonic stem cell markers, CD133, Bmi1, Sox2, Nanog and Oct4. Xenografts of MCF-7 and T47D Msi1 KD cells resulted in a marked reduction of tumor growth, reduced Msi1 and Notch1 expression and increased p21^CIP1 ^expression.

**Conclusion:**

Msi1 is a negative prognostic indicator of breast cancer patient survival, and is indicative of tumor cells with stem cell-like characteristics. Msi1 KD reduces tumor cell survival and tumor xenograft growth, suggesting that it may represent a novel target for drug discovery.

## Introduction

Breast cancer is the second most common malignancy and cause of cancer death among American women [1]. Despite advances in early detection, about 30% of patients with early-stage breast cancer have recurrent disease. Although systemic treatment of patients with chemotherapy, hormonal therapy and immunotherapy produce a high response rate initially, progression invariably occurs after a variable time interval [2]. This may result from a small subpopulation of cells, i.e., cancer 'stem' cells or tumor-initiating cells (TICs) that exhibit a high capacity for self-renewal, and thus contribute to tumor maintenance and metastasis [3], as well as relapse and drug resistance [4].

CD133 has been implicated as a TIC marker for malignancies of the lung [5], liver [6,7], colon [8,9], brain [10,11]. High CD133 expression has been noted in MCF-7 breast cancer cells that are either drug-resistant [12] or resistant to TRAIL-mediated apoptosis [13]. CD133^+ ^breast cancer cells form spheroid under low attachment conditions, and are enriched in stem cell markers, and rapidly form tumors in NOD/SCID mice [14]. Thus, by these criteria, CD133 may be considered a TIC marker. Another protein associated with breast TICs is Musashi1 (Msi1) [15-17]. Msi1 was first identified as a cell fate determinant for sensory organ development in *Drosophila *[18], and subsequent studies found the mammalian homolog to be a stem cell marker in the brain [19,20]. Msi1 is a highly conserved RNA-binding protein [21] that recognizes a (G/A)U_n = 1-3_AGU motif in the 3'-untranslated region of target mRNAs, such as Numb [22] and p21^CIP1^[23], as well as other genes involved in cell cycle regulation, proliferation and apoptosis [24]. When expressed in mammary epithelial cells, Msi1 activated a unique gene expression pattern associated with an autocrine pathway involving the growth factor Proliferin1 and inhibition of the secreted Wnt pathway inhibitor, Dickkopf3 [15,16]. This led to activation of Notch and Wnt signaling and mammary stem/progenitor cell proliferation.

The role of Msi1 in the etiology and progression of breast cancer is unknown. We hypothesized that Msi1 may promote breast TIC proliferation, and hereby influence survival outcome. Here we show that Msi1 is expressed in approximately two-thirds of primary breast cancers, and is associated with reduced survival. Msi1 was enriched in CD133^+ ^MCF-7 and T47D cells, particularly when grown as spheroid cultures, and Msi1 'knockdown' reduced spheroid colony formation and tumor xenograft growth. These results suggest that Msi1 regulates TIC proliferation and is a negative prognosticator for survival in breast cancer patients.

## Results

### Msi1 expression in breast cancer cell lines correlates with ErbB2

**Figure 1:**
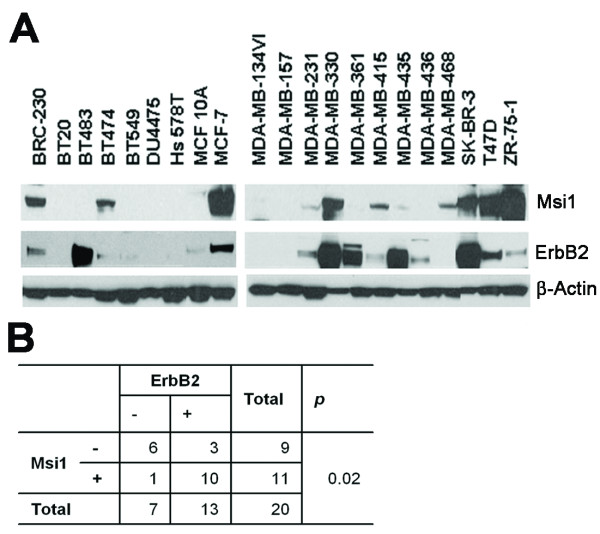
**Msi1 correlates with ErbB2 expression**. **A**. Msi1, ErbB2, ERα and β-actin were measured in 20 breast cancer cell lines and in immortalized breast epithelial cell line MCF-10A by western blotting. **B**. There is a statistically significant correlation between Msi1 and ErbB2 (P = 0.02) by Fisher's Exact test.

Twenty human breast cancer cell lines and one immortalized human mammary epithelial cell line (MCF-10A) were used for western analysis of Msi1 (Figure 1A). Eleven of 20 human breast cancer cell lines (55%) expressed Msi1, and 13 cell lines showed a strong correlation between Msi1 and ErbB2 (*P *= 0.02) (Figure 1B). There was no significant correlation between Msi1 and ERα (results not shown).

### Msi1 expression is increased in CD133^+ ^and tumor cells in spheroid culture

**Figure 2:**
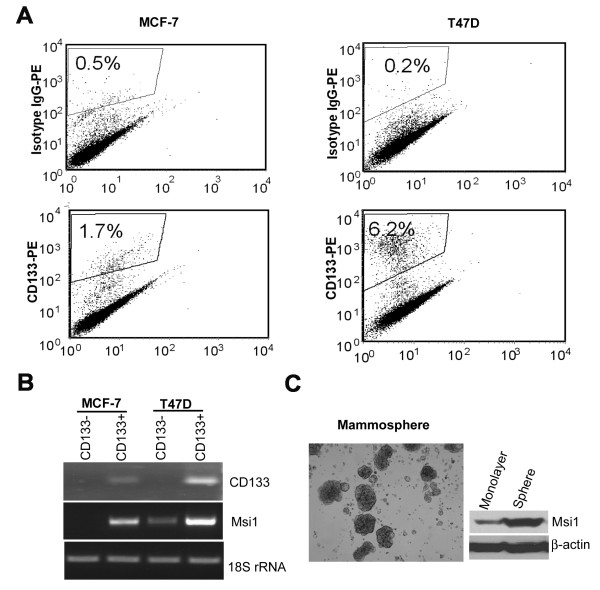
**Msi1 is enriched in breast tumor initiating cells**. **A**. MCF-7 and T47D cells contain a CD133^+ ^population. Cells sorted by FACS revealed that CD133^+ ^cells represented 1.7% and 6.2% of the total cell population in MCF-7 and T47D cells, respectively. **B**. Msi1 is expressed predominantly in CD133^+ ^cells. MCF-7 and T47D cells were sorted as in **A**, and CD133 and Msi1 were measured by RT-PCR. **C**, Spheroid cultures of MCF-7 cells are enriched in Msi1. Cells were grown for 3 weeks in ultra low attachment plates in DMEM media without serum and supplemented with growth factors. Spheroid colonies were harvested and Msi1 expression was determined by western blotting.

Both CD44^+^/CD24^- ^and CD133 have been used as TIC markers for breast cancer and other malignancies. Our results indicate that CD44^+^/CD24^- ^cells were very rare in T47D (0.04%) and MCF-7 (0.36%) cells (Additional file 1, Figure S1). In contrast, CD133 was expressed in 1.7% and 6.2% of MCF-7 and T47D cells, respectively (Figure 2A), and Msi1 mRNA was expressed predominantly in cells expressing CD133 mRNA (Figure 2B). MCF-7 cells grown in low attachment plates formed spheroid cultures, which were enriched for Msi1 expression in comparison to monolayer cells (Figure 2C).

### Msi1 KD in MCF-7 and T47D cells reduces CD133, ErbB2, pERK, Notch1 and ES cell markers and increases p21^CIP1^

**Figure 3:**
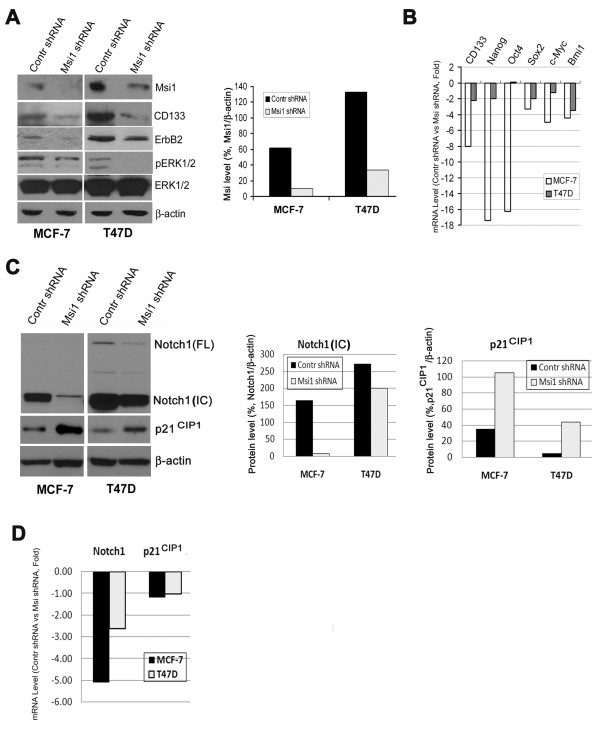
**Msi1 'knockdown' by a shRNA preferentially reduces CD133^+ ^cells and stem cell marker expression**. **A**. Lentivirus expression of an Msi1 shRNA. Left, 'Knockdown' (KD) of Msi1 in MCF-7 and T47D spheroid cells by an Msi1 shRNA (Msi1 shRNA) reduces Msi1, CD133, ErbB2 and pERK expression vs. the control shRNA (Contr shRNA). Right panel, quantitation of the western blot indicates that Msi1 was reduced by 77-84% in MCF-7 and T47D cells. **B**. Msi1 KD reduces stem cell marker expression in MCF-7 and T47D cells. CD133, Nanog, Oct4, Sox2, c-Myc and Bmi1 mRNA levels were determined by qRT-PCR. All stem cell markers, with the exception of Oct4 in T47D cells, were reduced by Msi1 knockdown. **C**. Left panel, Msi1 KD reduces Notch1 and increases p21^CIP1 ^in MCF-7 and T47D spheroid cells. IC, Notch intracellular domain; FL, full-length Notch1. Middle panel, quantitation of Notch1 expression. Right panel, quantitation of p21^CIP1 ^expression. **D**. Msi1 KD reduces Notch1 mRNA 2.7-5-fold, but not p21^CIP1^, in MCF-7 and T47D spheroid cells, respectively.

Lentivirus-mediated Msi1 KD was used to deplete Msi1 in MCF-7 and T47D cells grown in spheroid culture. After 3 weeks of selection, cells were harvested for western and RT-PCR analysis. Msi1 shRNA expression significantly reduced Msi1 levels in MCF-7 and T47D cells, and correlated with decreased CD133, ErbB2 and pERK1/2 expression (Figure 3A); Msi1 protein levels were reduced in MCF-7 and T47D cells by 83% and 77%, respectively, (Figure 3A, bar graph). qRT-PCR analysis confirmed reduction of CD133 mRNA in MCF-7 cells, as well as expression of the somatic and ES cell markers, Nanog, Oct4, Sox2, c-Myc and Bmi1 (Figure 3B). These changes, with the exception of Oct4, also occurred in T47D cells (Figure 3B). Msi1 KD reduced Notch expression at both the mRNA and protein levels and increased p21^CIP1 ^at only protein levels, and these effects were more pronounced in MCF-7 cells (Figure 3C, D).

### Msi1 KD reduces tumor spheroid cell colony formation

**Figure 4:**
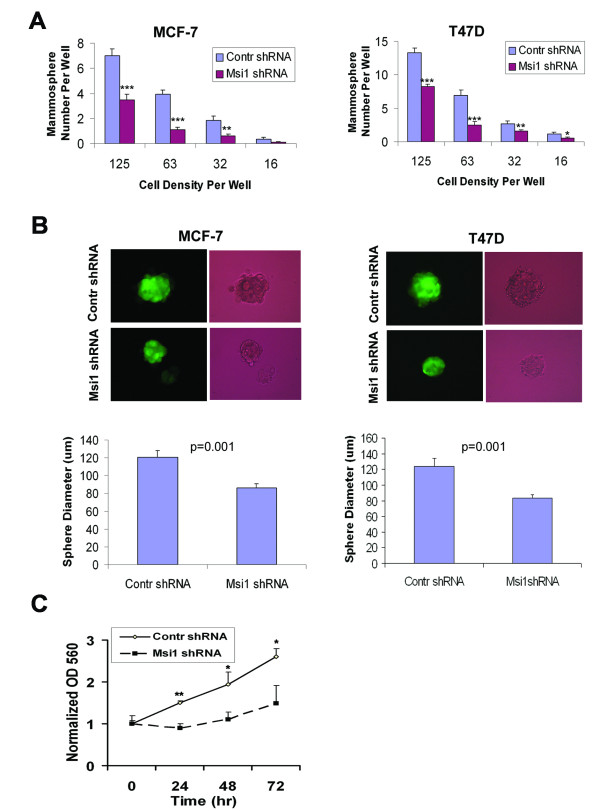
**Msi1 knockdown reduces spheroid colony formation**. MCF-7 and T47D cells were transduced with either an Msi1 shRNA (Msi1 shRNA) or control shRNA (Contr shRNA) as in Figure 3A. **A**. Cells were seeded at varying dilutions into 96-well ultra low attachment plates in DMEM media without serum and supplemented with growth factors, and the number of colonies determined after 3 weeks. Msi1 KD significantly reduces colony formation in MCF-7 and T47D cells (***P < 0.001; **P < 0.01; *P < 0.05; two-tailed Student's *t *test; N = 12). **B**. Msi1 KD reduces spheroid colony size. Upper panel, spheroid colony morphology by fluorescence (GFP) and Brightfield microscopy; Lower panel, colony diameter at a density of 25 cells/well. Msi1 KD significantly reduced colony size (*P *< 0.001; two-tailed Student's *t *test; N = 10). **C**. After 10 days in spheroid culture, MCF-7 cells were replated as monolayer cultures and cell growth was measured by sulforhodamine B staining at 24, 48 and 72 hr. Msi1 KD reduces the growth of MCF-7 cells 50% vs. control cells (Contr shRNA). Each value represents the mean ± S.E. of 4 samples (**P < 0.01, *P < 0.05 by the two-tailed Student's *t *test.

MCF-7 and T47D cells stably expressing either the control or Msi1 shRNA were seeded into 96-well ultra low attachment plates at limiting cell densities ranging from 125 to 16 cells per well in order to determine the self-renewal capacity of the TIC population [25] (Figure 4A). After 3 weeks of selection, the number of spheroid colonies was markedly reduced in the Msi1 shRNA-transduced cells (Figure 4A). Measurement of colony size, an indication of progenitor cell proliferation [25], at a cell density of 125 cells per well was reduced by 30% (*P *= 0.001) in both MCF-7 and T47D cells (Figure 4B). Dissociation of spheroid colonies into single cells resulted in reformation of the spheroid colonies, indicating that this phenotype was stable (results not shown). These data suggest that Msi1 modulates TIC proliferation to a greater extent than progenitor cell proliferation. When spheroid MCF-7 cells were dissociated with trypsin and replated in 96-well plates as monolayers, cell growth remained slower in cells after Msi1 KD than in control cells (Figure 4C).

### Msi1 KD inhibits the growth of breast tumor xenografts

**Figure 5:**
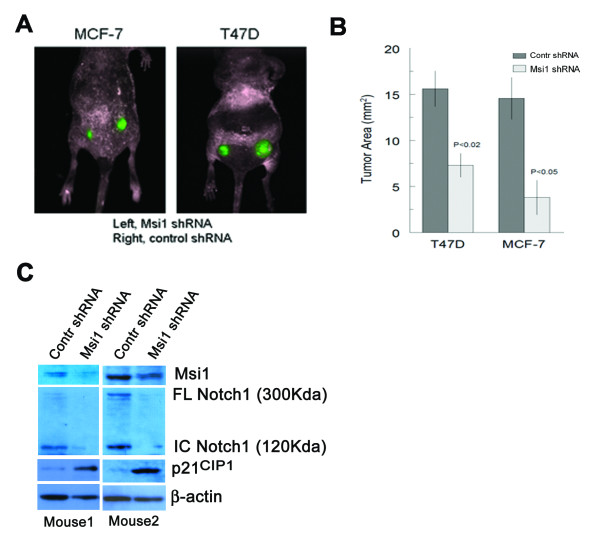
**Msi1 knockdown reduces breast tumor xenograft growth**. **A**. Fluorescent imaging of GFP expression in MCF-7 and T47D breast tumor xenografts in nude mice. Tumor cells transduced with either an Msi1 shRNA or control shRNA were transplanted into opposite flanks of each animal, and after 8 weeks tumor size was visualized with a Maestro imaging instrument. **B**. Reduction of tumor growth by Msi1 shRNA. Bar graphs indicate the relative changes in tumor xenograft area computed by pixel imaging. Each value represents the mean ± S.E. from five animals; P < 0.02 and P < 0.05 for T47D and MCF-7 xenografts, respectively, by the two-tailed Students *t *test. **C**. Msi1 KD in MCF-7 and T47D xenograft tumors reduces Msi1 and Notch1, and increases p21^CIP1 ^protein expression.

To assess tumor growth after Msi1 KD, spheroid cultures of T47D and MCF-7 cells expressing either the non-silencing or Msi1 shRNA were engrafted into nude mice at either 10,000 cells/site (T47D) or 100,000 cells/site (MCF-7) (Figure 5A). Msi1 KD in either cell line resulted in a 50-60% reduction in tumor growth (Figure 5B). Msi1 KD was confirmed in the tumors, and was associated with decreased Notch1 and increased p21^CIP1 ^protein expression (Figure 5C) as found for cells in culture (Figure 3C). Lower inocula of 1,000 T47D cells and 10,000 MCF-7 cells did not engraft, and may reflect an inadequate number of TICs in these cell lines.

### Msi1 expression in human breast cancers correlates inversely with survival and directly with metastasis

**Figure 6:**
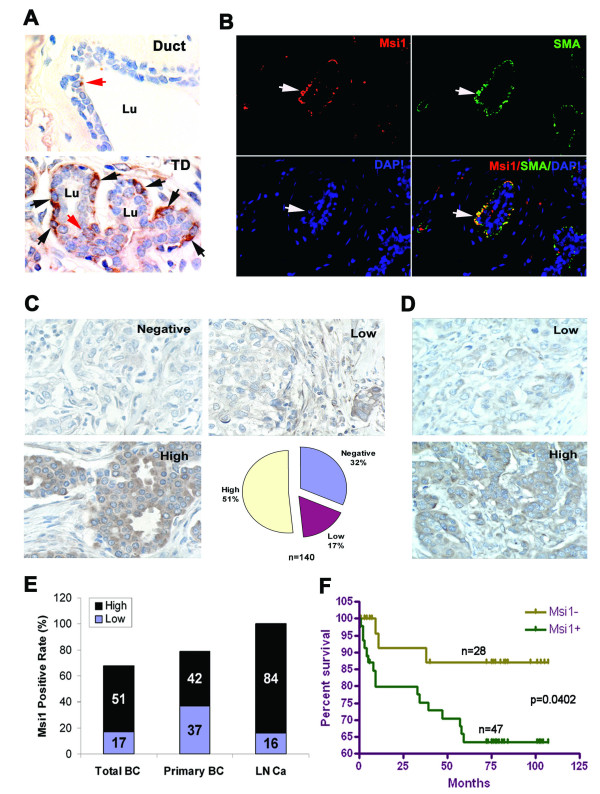
**Msi1 expression in breast cancer tissue microarrays**. **A**. Upper panel, Msi1 is rarely expressed in large ductal epithelial cells of normal breast tissue (red arrow), Lower panel, Msi1 is frequently expressed in terminal ductal myoepithelial cells (black arrows), whereas, one Msi1^+ ^cell is present in terminal ductal epithelial cells (red arrow). **B**. Msi1 and smooth muscle actin (SMA) are co-expressed in terminal ductal myoepithelial cells. **C**. Upper left panel, a breast cancer specimen "negative" for Msi1 expression (<1% positive cells). Upper right panel, a breast cancer specimen with "low" Msi1 expression (1-30% positive cells). Lower Left panel, a breast cancer specimen with "high" Msi1 expression (>31% positive cells). Lower right panel, the relative percentages of "negative", "low" and "high" Msi1 expression in all samples (N = 140). **D**. "Low" and "high" Msi1 expression in lymph node metastases. **E**. Msi1 is expressed in all metastatic breast cancer specimens. Bar graph indicates that 84% of lymph node metastases (LN; N = 19) had high Msi1 expression vs. 42% of matched primary tumors (Primary BC; N = 19) and 52% of all breast cancer specimens (Total BC; N = 140). **F**. Kaplan-Meier analysis indicates that a larger percentage of patients with Msi1^+ ^tumors (N = 47) survive <5 years vs. patients with Msi1^- ^tumors (N = 28). These results suggest that Msi1 is a negative prognostic marker.

The localization of Msi1 was examined in 26 adjacent normal breast tissues to determine if Msi1 expression was associated primarily with the terminal ductal lobulo-alveolar unit (TDLU). In large ducts located outside the TDLU, Msi1 was rarely present in either ductal epithelium (0.03%, 35/10722 cells) or myoepithelium (0.05%, 9/1902 cells). In the TDLU however, Msi1 was expressed in 10.6% (353/3321 cells) of myoepithelial cells and 1.2% (117/9545 cells) of terminal ductal epithelium (Figure 6A). To confirm the presence of Msi1 in myoepithelium, double immunofluorescence was used to measure the localization of Msi1 with the myoepithelial cell marker, smooth muscle actin (SMA) (Figure 6B). Msi1 and SMA co-localized, indicating the propensity of myoepithelial cells to express Msi1. To determine the significance of Msi1 expression in primary breast cancer, tissue microarrays of 140 tumors were analyzed by immunohistochemistry. Approximately two-thirds of the tumors were Msi1^+^, including 51% with high Msi1 and 17% with low expression (Figure 6C). Comparison of 19 lymph node metastases with their matched primary tumor revealed that Msi1 was highly expressing in 84% of metastases vs. 42% of matched primary tumors (Figure 6D, E). Analysis of 75 patients with a follow-up period ranging from 1 to 107 months revealed that 87% of patients with tumors lacking Msi1 survived >5 years vs. 62% of patients whose tumors were Msi1^+ ^(Figure 6F). Survival in patients with Msi1^+ ^tumors was 75.0 ± 6.2 months (mean ± SD) vs. 102.1 ± 4.8 in patients with Msi1- tumors (P = 0.040 by the log-rank test) (Figure 6F). Msi1 protein expression correlated with the 5-year survival events of breast cancer patients (P = 0.023) (Table 1). Analysis of the effect of Msi1 expression on survival time with or without adjustment for age, TNM stage, tumor grade, and tumor size in the Cox regression model indicated that the hazard role of Msi1 was significant. There was no statistically significant correlation between Msi1 expression and either TNM stage, tumor grade, ErbB2, ER, PR or p53 expression (results not shown). Thus, Msi1 expression is a prognosticator of TIC burden in breast cancer.

**Table 1:** Msi1 correlates with five year survival.

		Msi1			*P*
			
		Negative	Low	High	
Survival Year	>5	18	9	19	0.023
		
	<5	1	5	13	

Stage	0	0	0	1	0.446
		
	II	18	7	19	
		
	III	10	10	19	

Grade	I	3	0	4	0.126
		
	II	14	5	20	
		
	III	10	12	13	

## Discussion

Msi1 is an RNA-binding protein that functions as a translational repressor [20], and is widely used as a marker of stem cells in embryonic and adult tissues [18,21]. Although Msi1 has been reported to be elevated in high grade gliomas [26] and liver cancer [27], little is known about its function and prognostic value. Here we report that Msi1 is expressed in a high proportion of primary breast cancers and cell lines, particularly in metastatic disease. The most important prognostic factor influencing the outcome of patients with invasive breast cancer is whether the tumor has spread regionally or systemically [28], and there is evidence that TICs play an important role in mediating metastasis. Our finding that 84% of breast cancer lymph node metastases expressed Msi1, in comparison to 42% of matched primary tumors, is consistent with the role of TICs in metastatic disease. Since Msi1 predicted shorter survival as well, it may ultimately be a useful prognostic indicator of regional spread of the disease.

In normal breast tissue, Msi1 is co-localized predominantly with myoepithelial cells in the TDLU, where approximately 10% of the cells are Msi1^+^. Msi1 has been reported to be present in 0.6% of the total lobular epithelial cells, and enriched in label-retaining cells [17], which divide asymmetrically as stem cells [29]. Msi1-overexpressing mammary epithelial cells express stem and progenitor cell markers, where Msi1 co-localizes with CK14^+ ^myoepithelial cells [16], a phenotype consistent with the basal cell characteristics of mammary stem cells [30]. In the present study, Masi1 was functionally active in breast cancer cells, since inhibition of colony expansion and tumor growth by Msi1 KD resulted in increased Notch and reduced p21^CIP1^. Numb, a translational target of Msi1, promotes the degradation of intracellular Notch [22], and hence Msi1 KD would reduce Notch expression (Figures 3 and 5). p21^CIP1^, an inhibitor of cyclin-dependent kinases, is a translational target of Msi1 [23], and hence Msi1 KD would increase its expression and stimulate growth (Figures 3 and 5). These data indicate that Notch and p21^CIP1^, among other factors, regulate the TIC capacity of MCF-7 and T47D cells, and are consistent with the growth inhibitory activity of Msi1 KD in medulloblastoma [31] and colon tumor [32] cells.

CD44^+^/CD24^- ^and CD133 have been reported as TIC markers for breast cancer [5-11,13,14,36-38]. CD44^+^/CD24^- ^cells represented a very small percentage (0.02-0.36%) of MCF-7 and T47D cells [33] (Additional file 1, Figure S1). In contrast, CD133 was expressed in 1.7-6.2% of the cells, which were also enriched in Msi1. Msi1 was also enriched in spheroid cells, which have been reported to be less sensitive to ionizing radiation than monolayer cells and exhibit reduced senescence [34,35]. Mammary dysplasia induced by constitutively active hedgehog signaling also leads to an expansion of murine stem cells as high as 15-33% with an increased proliferative capacity [36]. Similar findings of an increased tumorigenic capacity have been reported for spheroid breast cancer cells in pleural effusions [37]. Thus, the capacity to form anchorage-independent spheroid cells reflects the TIC capacity of tumor cells, and is consistent with our tumor xenograft data.

In addition to affecting TIC proliferation, Msi1 regulated the expression of several somatic and ES cell markers, including CD133, Bmi1, Nanog, Oct4, Sox2 and c-Myc (Figure 3). Although, CD133 is TIC marker [5-11,13,14,38], its function in this context is largely unknown. Bmi1 is a polycomb protein that plays a critical role in somatic stem cell self-renewal based on gain-of-function and loss-of-function analyses [39,40]. Oct4, Sox2, Nanog and Lin-28 [41], as well as Oct3/4, Sox2, Klf4, and c-Myc [42] were shown to induce pluripotent stem cells from human fibroblasts. An ES cell gene signature has also been reported in poorly differentiated breast cancers and other tumors, and correlated with poor survival [43]. Thus, Msi1 appears to regulate transcriptional pathways required for survival and maintenance of the undifferentiated state.

Previously, Msi1 was found to activate an autocrine process in mammary epithelial cells that resulted in progenitor cell expansion through proliferin-mediated activation of ERK and Wnt and Notch signaling [15,16]. These findings are in agreement with the reduction of pERK1/2 and Notch1 expression after Msi1 KD. Wnt and ERK signaling constitute an oncogenic positive feedback loop [44], and inhibition of these pathways led to the differentiation of ES cells [16]. It therefore seems likely that activation of Wnt signaling through ERK by Msi1 plays a similar role in the maintenance of TIC proliferation and the undifferentiated state [15,16].

There was also a correlation between Msi1 and ErbB2 expression. ErbB2 was reduced in cells after Msi1 KD, suggesting that either ErbB2-expressing cells are only affected or that Msi1 acts downstream of ErbB2 signaling. ErbB2 is notable for its role in the pathogenesis of breast cancer and as a therapeutic target, and is frequently associated with metastatic disease and tumor progression [45]. Recently, ErbB2 was shown to induce Notch1 activity in breast cancer cells [46], which is consistent with its ability to drive mammary stem cell proliferation, tumorigenesis and invasion [47]. Since Msi1 activates the Notch pathway by directly targeting Numb, our findings suggest a common endpoint between the Msi1 and ErbB2 pathways that merits further examination in a larger subset of patients.

## Conclusion

Msi1 plays a role in the proliferation of breast TICs that denotes a more metastatic, and presumably, a therapy-resistant cell population that reflects poor outcome. Future studies will address if Msi1 may serve as a TIC-selective therapeutic target for breast cancer and other malignancies.

## Methods

### Cell Lines

Human breast cancer cell lines BRC-230, BT20, BT483, BT474, BT549, DU4475, Hs578T, MCF-7, MDA-MB-134VI, MDA-MB-157, MDA-MB-231, MDA-MB-330, MDA-MB-361, MDA-MB-415, MDA-MB-435, MDA-MB-436, MDA-MB-468, SK-BR-3, T47D and ZR-75-1, and immortalized human mammary epithelial cell line MCF-10A were obtained from the Tissue Culture Shared Resource, Lombardi Comprehensive Cancer Center. MCF-7 and T47D cells were maintained in Dulbecco's- modified Eagle's medium (DMEM) (Sigma-Aldrich Chemical Co., St. Louis, MO) supplemented with 10% fetal bovine serum at 37°C under 5% CO2 in a Sanyo CO_2 _incubator.

### Western Blotting

Cells lysates were prepared on ice [48], and protein concentration was determined using the Bicinchonic Acid Assay (Pierce, Rockford, IL). Cell lysates (20 μg protein) were separated in Criterion XT Bis-Tris Gels (Bio-Rad), transferred to a polyvinylidene difluoride membrane (Amersham Biosciences, Piscataway, NJ) and separated in the Criterion System (Bio-Rad, Hercules, CA). Proteins were detected with the appropriate primary antibody using horseradish peroxidase-linked secondary antibodies, and visualized by chemiluminescence with the West Pico system (Amersham Biosciences, Piscataway, NJ). Primary antibodies included rat anti-Msi1 or anti-biotinylated Msi1 (1:1000) [49] (14H-1, Dr. Hideyuki Okano, Keio University, Tokyo, Japan), rabbit anti-ErbB2 (1:1000) (Millipore, Danvers, MA), mouse anti-ERα (1:200, Santa Cruz Biotechnology, Santa Cruz, CA), rabbit anti-CD133 (1:1000) (Cell Signaling Technology, Danvers, MA), rabbit anti-pERK1/2 (1:1000)(Cell Signaling Technology), mouse anti-β-actin (1:5000) (Sigma-Aldrich Corp), rabbit anti-Notch1(D1E11) (Cell Signaling Technology) and mouse anti-p21^CIP1 ^(EMD Chemicals, Gibbstown, NJ).

### Flow Cytometry

For CD133, CD24 and CD44 detection, MCF-7 and T47D cells were suspended at a concentration of 0.5-1.0 × 10^6 ^cells/ml in ice-cold PBS containing 3% fetal bovine serum (PBS/FBS). One μg of CD133 unconjugated primary antibody (Cell Signaling Technology), CD24-biotin or CD44-phyoerythrin(PE)-Cy5 (eBioscience, Inc. San Diego, CA) or control IgG isotype antibody was incubated with 10^6 ^cells on ice for 60 min. Cells were washed three times by centrifugation at 400 × *g *for 5 min, and resuspended in ice-cold PBS/FBS. CD24 was measured with FITC-conjugated streptavidin (eBioscience) (1:100 dilution) after incubation for 45 min. CD133 was measured with an anti-rabbit secondary antibody conjugated with PE after incubation at room temperature for 45 min. Cells were sorted with a Becton Dickinson FACSort system into CD133^+ ^and CD133^- ^cell populations and analyzed with FCS Express V3 software (De Novo Software, Ontario, Canada). Cells were washed twice with PBS and the cell pellet stored at -80°C for RT-PCR.

### Lentivirus-mediated shRNA Expression

Msi1 expression was 'knocked down' using an shRNAmir GIPZ lentiviral vector targeting the sequence, 5'-CGT-CCT-GTA-TCA-TAT-GTA-AAT-3' in the 3'-UTR of Msi1 mRNA (Oligo ID # V2HS_280120; Open Biosystems, Huntsville, AL) [31]. TLA-HEK293T cells (Open Biosystems) were transfected with the Trans-Lentiviral Packaging Mix and pGIPZ transfer vector at 50% confluence using Arrest-In transfection reagent (Open Biosystems) according to the manufacturer's protocol. After incubation for 48-72 hr, the virus-containing supernatant was collected and centrifuged at 3,000 rpm for 20 min at 4°C, mixed 50:50 with fresh cell culture media, and used to transduce MCF-7 and T47D cells. Lentivirus expressing either a non-silencing control shRNA (shRNAmir, Open Biosystems) served as a negative control. Cells were selected for stable integration of the virus by incubation with 2.5 μg/ml puromycin (Sigma-Aldrich Corp.) for 10 days. The efficiency of integration was monitored by green fluorescent protein (*GFP*) co-expressed by the lentivirus.

### RT-PCR

RNA was extracted using an RNeasy mini kit (Qiagen, Valencia, CA) using the manufacturer's protocol. One μg RNA was reverse-transcribed in a total volume of 20 μl using the Omniscript RT kit (Qiagen). PCR was performed in triplicate using an ABI-Prism 7700 instrument (Applied Biosystems, Foster City, CA) and SYBR Green I detection (Applied Biosystems) according to the manufacturer's protocol. Amplification using the appropriate primers (Additional file 2, Table S1) was confirmed by ethidium bromide staining of the PCR products in an agarose gel. The expression of each target gene was normalized to the expression of 18S RNA and is presented as the ratio of the target gene to 18S RNA, expressed as 2^-ΔCt^, where Ct is the threshold cycle and ΔCt = Ct ^Target ^- Ct ^18S ^as described previously[16].

### Spheroid Cell Culture

Spheroid cell culture was carried out in ultra low attachment plates as described with small modifications [25]. Briefly, MCF-7 and T47-D cells growing as monolayer cultures were trypsinized with 0.05% trypsin-0.5 mM EDTA (Invitrogen, Carlsbad, CA), washed twice with PBS, counted and seeded into a 6-well plate at a density of 3,000 viable cells/ml or into a 96-well plate at serial dilutions of 125 to 16 cells per 200 μl. Cells were grown in serum-free DMEM medium supplemented with 1× B27 (Invitrogen), 20 ng/ml EGF (Sigma) and 20 ng/ml FGF-2 (Invitrogen) at 37°C under 5% CO_2_. Spheroid clusters were counted and collected by gravity or gentle centrifugation (800 *g*, 10 sec) after 21 days, and dissociated in 0.05% trypsin-0.5 mM EDTA for 10-15 min by gentle pipeting. Cells were filtered through a 40-μm nylon mesh sieve (Falcon), analyzed microscopically for single cellularity and counted. Successive passages were plated at 1,000 cells/ml in 6-well plates and colony size quantitated using MetaMorph software.

### Growth Assay

Cells were seeded in 96-well plates at 5,000 cells/well in 200 μl medium, and growth was determined 24 to 72 h later by sulforhodamine B staining [50]

### Tissue Microarrays

Breast cancer tissue microarray slides were obtained from Imgenex (San Diego, CA, Cat# IMH-371 and IMH-364), US Biomax (Rockville, MD, Cat#BR951) and Cybrdi (Xian, China, cat# CC08-01). There were a total of 140 cases of primary breast cancer, 19 metastatic lymph node carcinomas and 24 normal adjacent breast tissues. All patients were female between 20 and 84 years of age, with a median age of 47. Eighty four of 140 cases contained TNM stage data, 78 of 140 cases contained ER, PR and p53 status, and 40 of 110 cases were analyzed for ErbB2 expression (Table 1). After diagnosis, 75 of 140 patients were followed up for 1 to 107 months, with a median follow-up time of 76 months, and 11 were deceased by 60 months.

### Immunohistochemistry

Tissue microarray slides were baked at 62°C for 1 hr, deparaffinized in xylene for 15 min, and rehydrated in 100%, 95% and 70% ethanol for 5 min each. Antigen retrieval was achieved by steaming slides for 10 min in 10 mM citrate buffer, pH 6.0. Slides were washed three times in PBS and blocked for 1 hr in a buffer containing 10% goat serum in PBS, and incubated overnight at 4°C with a 1:1,000 dilution of rat anti-Msi1 antibody 14H-1 conjugated with biotin [49]. Slides were washed three times in PBS and antigen visualized with ABC Vectastain and DAB as substrate (Vector Labs, Burlingame, CA). Slides were counterstained with Harris-modified hematoxylin (Thermo-Fisher, Pittsburgh, PA) and mounted in Permount. The level of Msi1 expression in breast cancer tissue was categorized as negative, low (1~30% positive) and high (>31% positive). Four high power fields (400×) were randomly chosen for counting Msi1^+ ^cells in each core of the slides. The total cell number counted was ~1000 per core per patient. All slides were reviewed by one pathologist (Linnoila RI, MD) and one well-trained researcher in pathology (Wang XY, MD, PhD) blinded to the patients' clinical information. The agreement between the two researchers for the determinations of all tumor samples was 90.7% with a 0.76 of Cohen's Kappa indicating substantial agreement.

### Dual Label Immunofluorescence

Dual label immunofluorescence was performed using methods similar to those described previously [16]. The primary antibodies used were: mouse monoclonal anti-human smooth muscle actin (SMA) (clone 1A4, Dako North America, Inc. Carpinteria, CA) and rat monoclonal anti-mouse Msi1 antibody 14H-1 [49]. Antigens were retrieved as described above, and detected sequentially on sections by incubation for 1 hr with the first (Msi1) primary antibody followed by incubation with the appropriate PE-conjugated streptavidin (eBioscience, Inc), and then incubation for 1 hr with the second primary antibody followed by FITC-conjugated secondary antibodies. All incubations were at room temperature and sections were washed in PBS (3 × 5 min) between each step. Sections were mounted in an anti-fading reagent with 4',6-diamidino-2-phenylindole (DAPI) (Invitrogen). Control slides were included in each analysis in which non-immune serum was substituted for primary antibodies and secondary antibodies individually.

### Tumor Xenografts

MCF-7 and T47D cells were grown as spheroid cultures and transduced with either the non-silencing shRNA or Msi1 shRNA as described above. Ten athymic *nu/nu *female mice of 6 weeks of age were each injected s.c. with a 60-day release pellet containing 0.72 mg 17-β-estradiol. Five animals were then injected s.c. into opposite flanks with either 100,000 MCF-7 cells transduced with a non-silencing shRNA or MCF-7 cells transduced with the Msi1 shRNA mixed with 0.45 mg Matrigel (BD Biosciences, Inc.). The same protocol was used for xenografts of T47D cells except that the inoculum was 10,000 cells. The frequency of engraftment of MCF-7 and T47D cells at these inocula was approximately 80%, which is similar to what is observed for standard xenograft procedures using inocula of ~5 × 10^6 ^cells. After 8 weeks, tumor size was imaged for GFP fluorescence using a Maestro imaging system (Cambridge Instruments, Inc.) with 1-3% isofluorane in oxygen as anesthetic.

### Statistics

Fisher's exact test was used to analyze the correlation between Msi1 and ErbB2 protein expression in breast cancer cell lines. Fisher's exact two-tailed test was also used to determine the association between Msi1 and the clinic stage, grade and 5-year survival events in breast cancer patients. Student's *t *test was used to determine the significance of the differences between cell growth, spheroid cell number and sphere diameters in cell lines, as well as tumor size resulting from Msi1 KD. The Kaplan-Meier method, log rank test and Cox regression model were used to evaluate the effects of Msi1 protein expression on the overall survival of patients with breast cancer. All tests were two-sided and determined with SAS software (version 9.1; SAS Institute, Cary, NC, USA). *P *< 0.05 was considered statistically significant.

## Competing interests

The authors declare that they have no competing interests.

## Authors' contributions

XYW designed research, carried out the molecular and cell studies, analyzed data, wrote the paper, and LP contributed to the design of the shRNA studies, HY contributed to the xenograft studies, RIL contributed to the IHC slide review and analysis of clinical data, JL contributed to the tissue microarray studies, HO contributed to the IHC studies, and RIG contributed to the conception, design, analysis and interpretation of data. All authors read and approved the final manuscript.

## Author Details

Department of Oncology, Georgetown University, and Lombardi Comprehensive Cancer Center, Washington, DC 20007 (RIG); Cell and Cancer Biology Branch, Center for Cancer Research, National Cancer Institute, National Institutes of Health, Bethesda, MD 20892 (XYW)

## Supplementary Material

Additional file 1 **Figure S1: CD44 and CD24 expression in MCF-7 and T47D cells**. **A**, Flow cytometry for CD44 and CD24 in MCF-7. *Left panel*, IgG isotype control; *Right panel*, CD44/CD24 positive cells. **B**. Flow cytometry for CD44 and CD24 in T47D. *Left panel*, IgG isotype control; *Right panel*, CD44/CD24 positive cells. Click here for file

Additional file 2 **Table S1: List of primer sequences for qRT-PCR**. Click here for file
