# Supplementary figures and images for: Musashi1 regulates breast tumor cell proliferation and is a prognostic indicator of poor survival
Source: Mol Cancer. 2010 Aug 21;9:221. doi: 10.1186/1476-4598-9-221 (PMC2939568; doi:10.1186/1476-4598-9-221)

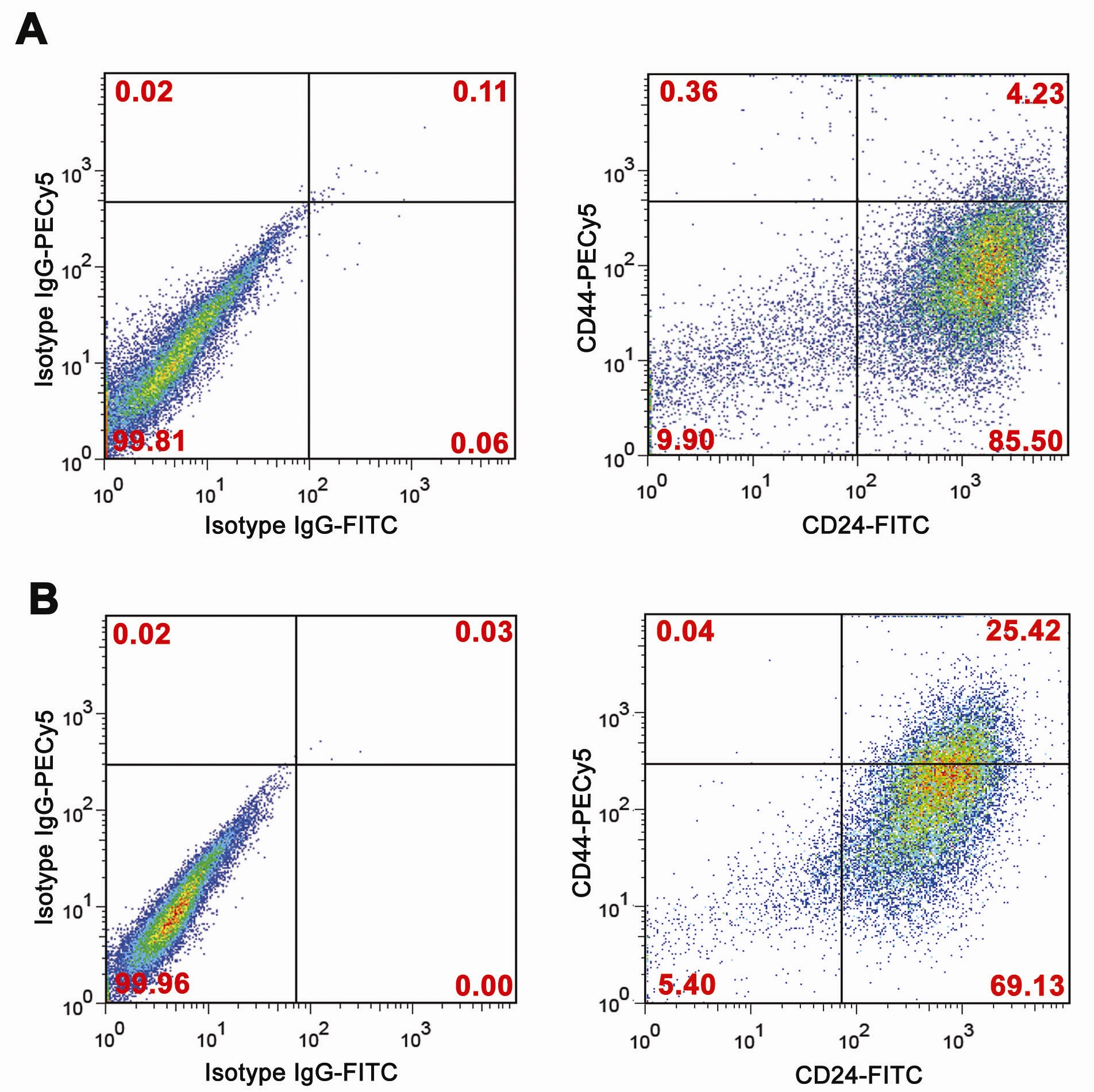

Supplement: Additional file 1 — Figure S1: CD44 and CD24 expression in MCF-7 and T47D cells. A, Flow cytometry for CD44 and CD24 in MCF-7. Left panel, IgG isotype control; Right panel, CD44/CD24 positive cells. B. Flow cytometry for CD44 and CD24 in T47D. Left panel, IgG isotype control; Right panel, CD44/CD24 positive cells. [file 1476-4598-9-221-S1.JPEG]
